# Effects of Luteolin and Quercetin in Combination with Some Conventional Antibiotics against Methicillin-Resistant *Staphylococcus aureus*

**DOI:** 10.3390/ijms17111947

**Published:** 2016-11-22

**Authors:** Muhammad Usman Amin, Muhammad Khurram, Taj Ali Khan, Hani S. Faidah, Zia Ullah Shah, Shafiq Ur Rahman, Abdul Haseeb, Muhammad Ilyas, Naseem Ullah, Sahibzada Muhammad Umar Khayam, Marcello Iriti

**Affiliations:** 1 Department of Microbiology, Kohat University of Science and Technology, Kohat 26000, Pakistan; pharmacyst2000@yahoo.com (M.U.A.); microbiologist63@yahoo.com (T.A.K.); 2 Department of Pharmacy, Shaheed Benazir Bhutto University, Sheringal 18000, Pakistan; shafiq@sbbu.edu.pk; 3 Department of Microbiology, Faculty of Medicine, Umm Al Qura University, Makkah 21955, Saudi Arabia; hanifaidah@gmail.com; 4 Department of Microbiology & Biotechnology, Sarhad University of Science and Information Technology, Peshawar 25000, Pakistan; zia.biotech@suit.edu.pk (Z.U.S.); milyas_1978@hotmail.com (M.I.); 5 Department of Clinical Pharmacy, College of Pharmacy, Umm Al Qura University, Makkah 21955, Saudi Arabia; amhaseeb@uqu.edu.sa; 6 Department of Pharmacy, Sarhad University of Science and Information Technology, Peshawar 25000, Pakistan; naseem.fls@suit.edu.pk (N.U.); umar.sahibzada@gmail.com (S.M.U.K.); 7 Department of Pharmacology, Khyber Medical University Institute of Medical Sciences, Kohat 26000, Pakistan; 8 Department of Agricultural and Environmental Sciences, Milan State University, 20133 Milan, Italy

**Keywords:** flavonoids, antibiotic resistance, MRSA, combination therapy, synergism, phytochemicals

## Abstract

The present study was designed to evaluate the effects of flavonoids luteolin (L) and quercetin + luteolin (Q + L) in combination with commonly used antibacterial agents against methicillin-resistant *Staphylococcus aureus* (MRSA) clinical isolates and *S. aureus* (ATCC 43300). Minimum inhibitory concentrations (MICs) of L and Q + L, as well as the MICs of flavonoids in combination with antibiotics were determined and results showed an increased activity of flavonoids with antibiotics. The synergistic, additive, or antagonistic relationships between flavonoids (L and Q + L) and antibiotics were also evaluated, and additive and synergistic effects were observed for some antibiotic + flavonoid combinations. In addition, some combinations were also found to damage the bacterial cytoplasmic membrane, as assessed through potassium leakage assay. The effects of flavonoids and flavonoids + antibiotics on *mecA* gene mutations were also tested, and no functional variation was detected in the coding region.

## 1. Introduction

Methicillin-resistant *Staphylococcus aureus* (MRSA) strains first became clinically relevant in 1968 when, in a Boston hospital, second-generation penicillin was ineffective as an antibiotic agent [1]; since then, reports on MRSA have been continuously rising. This issue is a global risk in developed countries such as Japan and Korea, which have a frequency of MRSA from 0% to around 70%, while increases of 40% in the UK and USA, and of 30% in Belgium, have been observed. However, this condition is not only confined to developed countries, and the situation is even worse in developing countries such as Pakistan, where the prevalence of MRSA is rapidly increasing, especially in the last decade. It has also been observed that MRSA incidence varies from region to region and even in different hospitals within the same region. In 1999, less than 50% of *S. aureus* clinical isolates from the intensive care unit in National Nosocomial Infection Surveillance system were found to be MRSA strains; in the next five years, this peaked to 63%, with reports of co-resistance for antibiotics such as Gentamycin, Ciprofloxacin, Rifampicin, Cotrimoxazole, Tetracycline, and Clindamycin [2]. A 10-month multicenter study revealed a 42% frequency of MRSA strains in *S. aureus* isolates, which is very alarming [3].

Flavonoids have been found to be effective against Gram-positive bacteria [4]. In a study on naringenin, apigenin, quercetin, kaempferol, and 7-*O*-butyl naringenin against *S. aureus* strains isolated from meat, all the compounds exhibited bacteriostatic activity. Quercetin was found to be the most effective, while electron microscopy analysis revealed bacterial cell wall damage by 7-*O*-butyl naringenin [5]. In another study, propolis obtained from two different sources was evaluated against clinical isolates and standard MRSA strains. The antibacterial activity of propolis was attributed to three flavonoids, in which pinobanksin-3-*O*-acetate and pinocembrin were reported to be effective against MRSA [6]. Similarly, in a separate study, luteolin, quercetin, chlorogenic acid, apigenin, and scutellarin were reported for their antibacterial activity against 29 MRSA clinical isolates [7].

Due to the rapidly growing resistance in *S. aureus*, this study was designed to evaluate the anti-MRSA effects of luteolin and quercetin alone and in different combinations with conventional antibiotics, in order to seek alternative therapeutic options to cope with antibiotic resistance and face the threat posed by this bacterium.

## 2. Results and Discussion

Antibiotic resistance in *S. aureus* is a global phenomenon and is constantly increasing due to the indiscriminate use of antibiotics, especially in developing countries such as Pakistan [8]. This has hastened the search not only for new and effective therapeutic agents, but also for making already blunted antibiotics effective again—the latter is a very handy option since new drug discovery requires huge investments and, generally, a very long time. There are many reports on the activity of flavonoids against *S. aureus* [8]. For instance, *Halostachys caspica* is known to contain seven different flavonoids including luteolin, which were evaluated for their activity against different bacterial strains such as *Escherichia coli*, *Pseudomonas lachrymans*, *Xanthomonas vesicatoria*, *S. aureus*, *S. haemolyticus*, and the fungus *Mangnaprothe oryzae*. Luteolin was found to be effective against all the tested strains [9]. Similarly, in another study, luteolin showed activity against MRSA [10]. Virulence factors of *S. aureus* represent an important aspect in pathogenesis, toxins in particular. Luteolin decreased α-toxin production by *S. aureus* and exhibited activity against MRSA isolates [11], in agreement with previous reports [12,13].

In another study on flavonoids, catechins, (−) epigallocatechin gallate (EGCG), and (−) epicatechin gallate (ECG) were associated with a decrease of MICs when used in combination with antibiotics [14]. A study exploring the mechanism of action of EGCG showed that interference in the biosynthesis of bacterial cell wall by binding with the peptidoglycan layer was the probable cause of bacterial inhibition. Moreover, ECG was found to generate lipoteichoic acids, thus causing changes in the chemical structure of teichoic acid within the bacterial cell wall [15]. Consequently, β-lactams such as ampicillin and amoxicillin more effectively damaged the bacterial cell wall. However, two other non-galloylated catechins from green tea extract, i.e., epigallocatechin (EGC) and (−)-epicatechin (EC), were not able to reduce bacterial resistance to β-lactams; they produced a synergistic effect when used along with EGCG and ECG. Therefore, these non-galloylated catechins were suggested to increase the galloylated catechin (EGCG and ECG) binding capacity to the bacterial cell membrane, thus suggesting their probable combined action. Moreover, non-galloylated catechins also increased oxacillin sensitivity against MRSA [16]. These studies suggest the complex interactions between flavonoids and bacterial cell.

**Figure 1:**
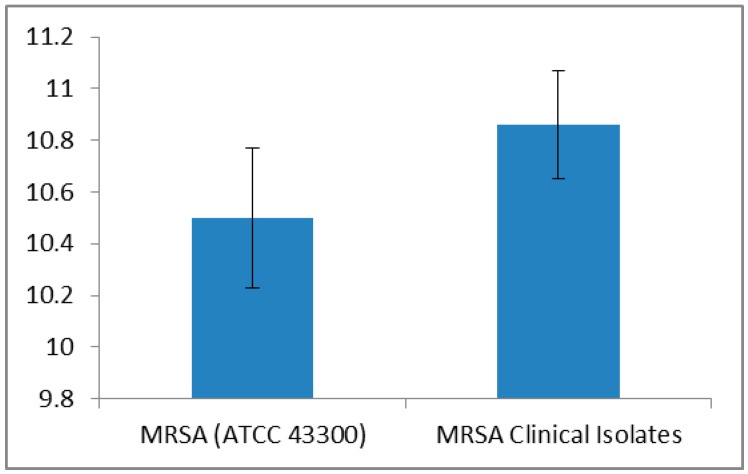
Average zone of inhibition (mm ± standard deviation) of luteolin against MRSA (methicillin-resistant *Staphylococcus aureus*) ATCC 43300 strain and MRSA clinical isolates (*n* = 100).

Different concentrationsof luteolin were tested for their activity against MRSA, and 600 µg was the effective concentration producing zone of inhibitions >10 mm (Figure 1), i.e., 10.8 ± 0.2 and 10.5 ± 0.2 mm against MRSA clinical isolates and ATCC 43300 strain, respectively.

**Figure 2:**
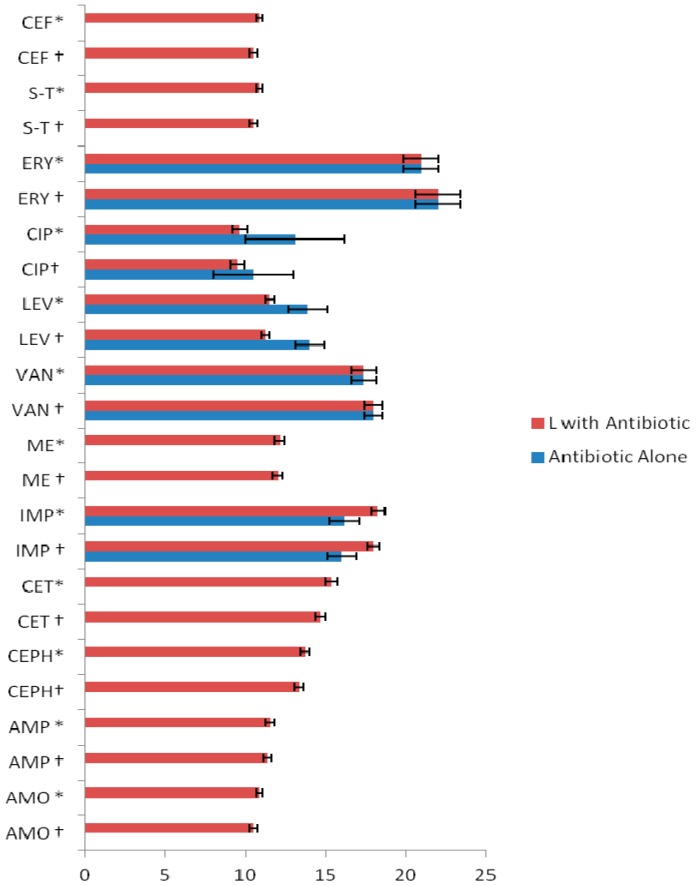
Average zone of inhibition (mm ± standard deviation) of luteolin (L) in combination with antibiotics against * MRSA clinical isolates (*n* =100); † MRSA ATCC 43300 strain. AMO = amoxicillin; AMP = ampicillin; CEPH = cephradine; CET = ceftriaxone; IMP = imipenem; ME = methicillin; VAN = vancomycin; LEV = levolfloxacin; CIP = ciprofloxacin; ERY = erythromycin; S-T = sulfamethoxazole-trimethoprim; CEF = cefixime.

In addition, luteolin increased the activity of conventional antibiotics. The highest enhancing effect was observed for IMP, and inhibitory zones were also recorded for other antibiotics like AMO, CEPH, ME, and CET. However, the activities of CIP and LEV were reduced. This is an interesting finding and, currently, we are investigating the principal mechanism(s) involved, such as physicochemical interaction between flavonoid and CIP or LEV, or other interactions with different molecular sites. A comparison between the activities of antibiotics and luteolin + antibiotics against standard MRSA strain and MRSA clinical isolates is shown in Figure 2.

Luteolin showed an antimicrobial enhancing effect on CET, higher than on AMP, ME, and CEPH; however, it had no effect on AMO. In general, these ineffective antibiotics acquired activity against both MRSA clinical isolates and the standard strain when combined with luteolin. The zones of inhibition of AMP increased from inactivity (≤6 mm) to 11.3 ± 0.2 mm against ATCC 43300 in combination with luteolin, while, against the clinical isolates, the inhibitory zones increased to 11.5 ± 0.2 mm. In the case of inactive CEPH, combination with luteolin produced inhibitory zones of 13.3 ± 0.3 mm and 13.7 ± 0.2 mm against ATCC 43300 strain and clinical isolates, respectively. The inhibition zones for CET + L were 14.6 ± 0.3 mm and 15.3 ± 0.3 mm against standard strain and clinical isolates, respectively. IMP was the only antibiotic active against MRSA, which showed a further increase in activity with luteolin, as inhibition zones increased from 16.0 ± 0.9 to 18.0 ± 0.4 and from 16.1 ± 0.4 to 18.2 ± 0.4 mm against reference standard and clinical strains, respectively. The change in the activity of antibiotics, in combination with luteolin, was found to be significant (*p* < 0.001). Similarly, the efficacy of different concentrations of quercetin, alone and in combination with antibiotics, has been previously reported against the *S. aureus* ATCC 43300 strain and its clinical isolates [17].

The results from the preliminary screening were further quantified by MIC assays, in which an effective concentration of 500 µg/mL luteolin was used. This concentration was in agreement with an earlier report [10], in which 38 flavonoids including 500 µg/mL luteolin were tested for their activities against MRSA, vancomycin-resistant Enterococci (VRE), *Klebsiella pneumoniae*, and *Staphylococcus epidermidis*. However, only luteolin was found to inhibit MRSA. Luteolin was also studied for its synergistic activity with AMO and was found to significantly reduce the viability of bacteria compared to luteolin and AMO alone [18]. In this context, luteolin was used in combination with quercetin as well as conventionally used antibiotics.

**Figure 3:**
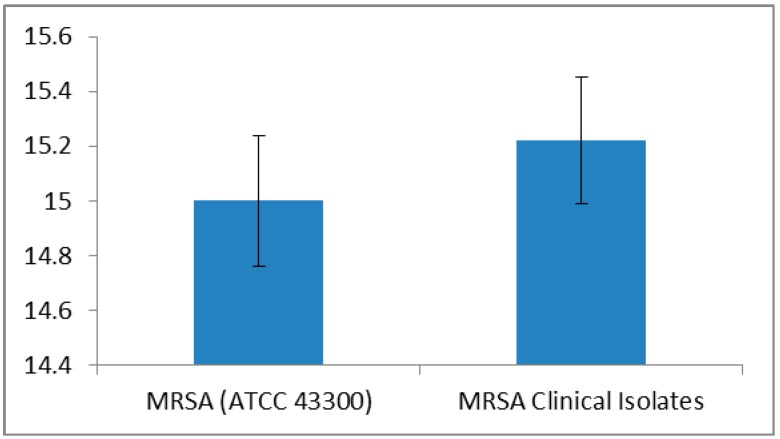
Average zone of inhibition (mm ± standard deviation) of luteolin + quercetin against MRSA (methicillin-resistant *Staphylococcus aureus*) ATCC 43300 strain and MRSA clinical isolates (*n* = 100).

The combination L + Q was tested for activity against MRSA clinical isolates and *S. aureus* ATCC 43300 strain. This combination showed a higher efficacy than the individual compounds (Figure 3).

**Figure 4:**
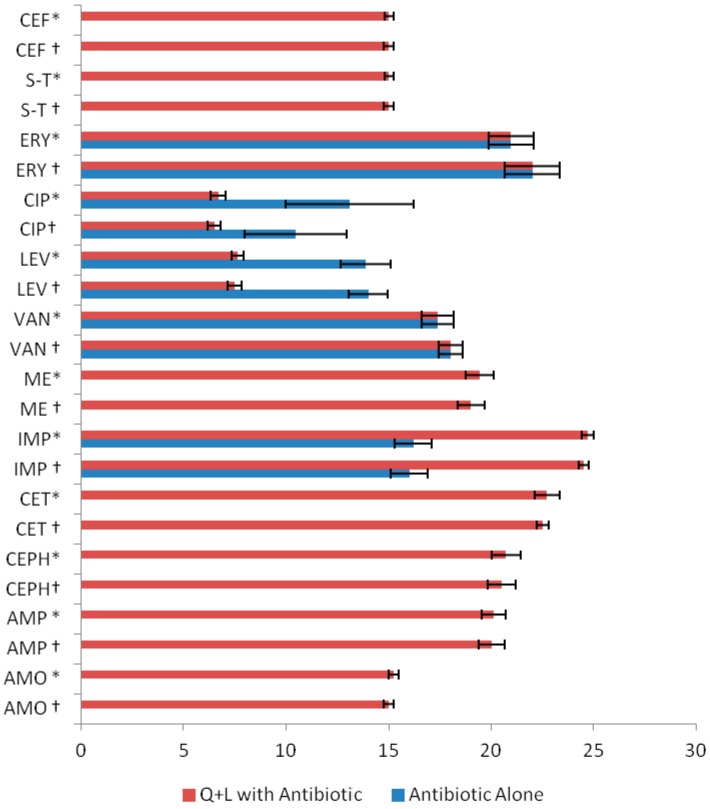
Average zone of inhibition (mm ± standard deviation) of quercetin (Q) + luteolin (L) in combination with antibiotics against * MRSA (methicillin-resistant *Staphylococcus aureus*) clinical isolates (*n* = 100) and † MRSA ATCC 43300 strain. (*p* < 0.05 for IMP; *p* < 0.001 ME, CET, CEPH, AMP, AMO).

The zones of inhibition observed with L + Q were 15.0 ± 0.2 mm against ATCC 43300 strain and 15.2 ± 0.2 mm in case of clinical isolates. Antibiotics improved their anti-MRSA activity when combined with L + Q, as reflected in the significant increase (*p* < 0.01) in inhibition zones compared to their individual inhibitions (Figure 4). The highest increase in activity was for CET with L + Q combination for MRSA ATCC 43300 strain (22.5 ± 0.2 mm), as well as MRSA clinical isolates (22.7 ± 0.6 mm); notably, this antibiotic was previously inactive. Pure ethanol was used to prepare stock solutions of L (15 g/L) and Q (10 g/L), which were then used in disk diffusion assays and in broth macrodilution MIC assays. In both assays, ethanol had no impact on the growth of bacteria (standard ATCC 43300 strain and clinical isolates), probably due to the evaporation of ethanol in a disk diffusion assay following the application of a test concentration on sterile blank disks, and due to dilution in MIC assays.

Similarly, activity increased for AMP (20.0 ± 0.64 and 20.1 ± 0.6 mm), CEPH (20.5 ± 0.6 and 20.7 ± 0.7 mm), and ME (19.0 ± 0.6 and 19.4 ± 0.6 mm) against MRSA (ATCC 43300 strain and clinical isolates, respectively). Q + L in combination enhanced IMP activity, from 16.0 ± 0.9 and 16.1 ± 0.9 to 24.5 ± 0.25 and 24.72 ± 0.29 mm against ATCC 43300 strain and clinical isolates, respectively. The IMP inhibition zones were greater than the CET ones, though, interestingly, the overall increase in activity of CET was higher than that of IMP, as the former rose from inactivity to 22.5 ± 0.2 mm, while the latter increased from 16.0 ± 0.9 to 24.5 ± 0.2 mm against ATCC 43300 strain. L + Q had no effect on AMO and SULPH-TRI, while the combination showed negative effect on CIP and LEV activities (Figure 4).

The MIC of luteolin was 500 µg/mL against ATCC 43300 strain and 39 clinical isolates, whereas it was 1000 µg/mL against the remaining isolates (*n* = 61). The exact MIC of L by incremental increase approach against clinical isolates (*n* = 61) was in the range of 500–540 µg/mL and averaged 516.83 ± 15.67 µg/mL against the clinical isolates, while for ATCC 43300 strain it was 500 µg/mL (Table 1).

**Figure 5:**
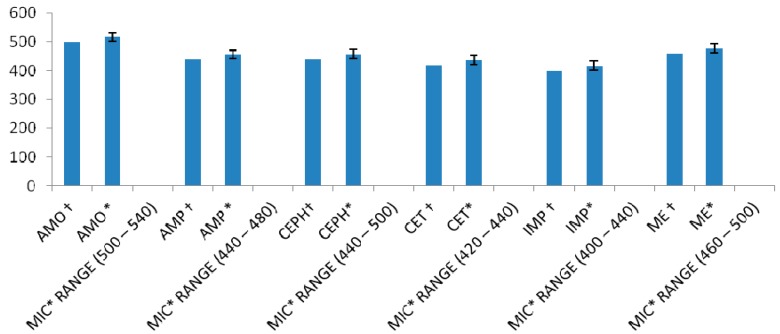
Exact minimum inhibitory concentrations (MIC, µg/mL) by incremental increase approach of luteolin in combination with antibiotics (µg/mL) against * MRSA clinical isolates (*n* = 100) and † MRSA ATCC 43300 strain; results are expressed as mean ± standard deviation. (*p* < 0.001).

The MIC of L was further reduced in combination with antibiotics, thereby exhibiting an increase in its activity (Figure 5). The highest decrease in MIC of L was observed with IMP for ATCC 43300 strain and clinical isolates, compared to other test antibiotics. The MIC of L alone was 500 µg/mL against ATCC 43300 strain, which decreased to 400 µg/mL when combined with IMP; similarly, against clinical isolates, MIC of L improved from 516.83 ± 15.67 to 417 ± 14.95 µg/mL.

A comparison between the MICs of L alone (Table 1) and in combination with antibiotics against ATCC 43300 strain and clinical isolates (Figure 5) indicated that the MIC reduction of L was nearly the same in its combination with AMP and CEPH, as the MIC of L improved from 500 to 440 µg/mL against ATCC 43300; conversely, MIC reduction was higher when rutin was combined with IMP and CET. The MIC of L with CET decreased to 420 and 437.20 ± 15.56 µg/mL against standard strain and clinical isolates, respectively (*p* < 0.001).

The MIC of L was 400 µg/mL when combined with Q against the standard strain (Table 2). Similarly, in the case of 42 clinical isolates, the MIC of L in combination with Q was 400 µg/mL, whereas, against the remaining 58 clinical isolates, it was 800 µg/mL. The same trend was observed for Q, as its MIC decreased from 300 to 200 µg/mL against ATCC 43300 strain, while, against clinical isolates, MIC was 200 µg/mL (*n* = 42) and was 400 µg/mL (*n* = 58).

The MIC of L + Q was also measured through the incremental increase approach to define the inhibitory concentrations of flavonoid combination against ATCC 43300 strain and the clinical isolates. The results (Table 2) showed that the MIC of L and Q in combination against ATCC 43300 were 380 and 200 µg/mL, respectively. The average MIC of L in combination with Q against 100 clinical isolates was 394.06 ± 13.75 µg/mL, while the MIC of Q was 215.04 ± 12.72 µg/mL. From the results in Table 2, the MIC of L in combination with Q decreased from 500 to 380 µg/mL against ATCC 43300, while, against clinical isolates, the average MIC was 394.06 ± 13.75 µg/mL, lower than the average MIC (516.83 ± 15.67 µg/mL) when L was used alone. Similarly, the MIC of Q improved to 200 µg/mL for ATCC 43300 and, in the case of clinical samples, the average MIC decreased from 279.00 ± 14.65 to 215.04 ± 12.72 µg/mL, which is suggestive of some synergism or additive relationship between flavonoids.

From the results reported in Table 3, it may be concluded that, in Q + L combination, the MIC of both flavonoids improved further when they were combined with antibiotics in comparison to Q + L alone, which is indicative of their increased activity against MRSA. For instance, the average MIC of L in Q + L combination with AMP decreased from 500 to 380 µg/mL, while the MIC of Q in this combination decreased from 260 to 200 µg/mL for the ATCC 43300 strain. A similar trend of reduction (*p* < 0.01) was observed in the MICs of flavonoids when used with other antibiotics. The MIC reduction of antibiotics with flavonoids was in the following order: Antibiotics + Q + L > Antibiotics + Q > Antibiotics + L.

In combination with flavonoids, all antibiotics facing resistance exhibited activity against MRSA, especially the cell wall synthesis inhibitors.

**Figure 6:**
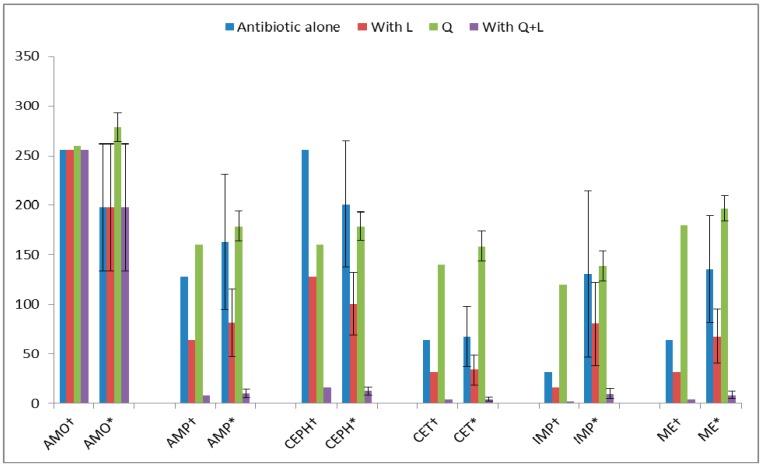
Minimum inhibitory concentration (MIC, µg/mL) of antibiotics and combination between antibiotics and luteolin (L) or quercetin + luteolin (Q + L) against * MRSA clinical isolates (*n* = 100) and † MRSA ATCC 43300strain; results are expressed as mean ± standard deviation (*p* < 0.001). Results for Q alone have been previously reported [13].

As shown in Figure 6, the MIC against ATCC 43300 strain decreased from 128 µg/mL, for AMP, to 64 µg/mL in AMP + L, whereas, against clinical isolates, the average MIC dropped from 162.85 ± 68.05 µg/mL, for AMP, to 81.43 ± 34.02 µg/mL in AMP + L. In combination with Q + L, the MIC of AMP decreased to 16 µg/mL for ATCC 43300 and its average MIC with Q + L decreased to 12.56 ± 3.98 µg/mL for the clinical isolates. The MIC of CEPH was 256 µg/mL against ATCC 43300 and average MIC was 200.96 ± 63.69 µg/mL for clinical isolates, while the average MIC of CEPH with Q + L was 50.38 ± 17.92 µg/mL for the clinical isolates. The reduction in the MIC of CEPH with Q + L (16 µg/mL) was higher than that of L (128 µg/mL) against ATCC 43300 strain. The MIC of CET was 64 µg/mL for ATCC 43300, but, in combination with Q, L and Q + L, the MICs dropped to 8, 32, and 4 µg/mL, respectively. The MIC of IMP + L against ATCC 43300 was 16 µg/mL, while, against the clinical isolates, the average MIC decreased to 80.16 ± 42.03 µg/mL. The MIC of IMP with Q + L against ATCC 43300 was 4 µg/mL, half that with L. Therefore, the reduction caused by Q + L in the MIC of IMP was higher than the reduction by L. The results revealed that the MICs of IMP decreased to a much higher extent when combined with flavonoids (*p* < 0.001). The reason for determining the MICs of these antibiotics alone and in combination with flavonoids was to ascertain whether synergistic or additive relationships exist between them.

It has been previously reported that catechins transform resistance of β-lactams in *S. aureus* by changing the cell membrane biophysical properties and affecting the movement of molecules across the cell membrane, as they compromise the functions of proteins embedded in the bilayer [19]. Synergism between amoxicillin and luteolin was previously reported against amoxicillin-resistant *E. coli*. The results revealed that amoxicillin and luteolin, in combination, decreased the number of amoxicillin-resistant *E. coli* cells and altered the permeability of the bacterial cell membrane [18]. Therefore, this study was designed to evaluate the potential of luteolin in combination with quercetin and other conventional antibiotics, especially cell wall inhibitors, against MRSA.

To establish whether a synergistic, additive, or antagonistic relationship existed between flavonoids and antibiotics, FICIs were determined. Data from Table 4 indicate a synergistic interaction between Q + L + CET and Q + L + IMP, while an additive relationship was found for the rest of the combinations.

In our previous study, an additive effect [13] was observed for quercetin in combination with antibiotics, with FICI <1. In the present report, additive effect is also found for Q + L with AMP, CEPH and MET (FICI ≤ 1). Interestingly, combination Q + L has a synergistic effect with IMP and CET (FICIs ≤ 0.5). Our findings are in agreement with previous reports [20,21] showing either additive or synergistic relationships between flavonoids and antibiotics.

**Figure 7:**
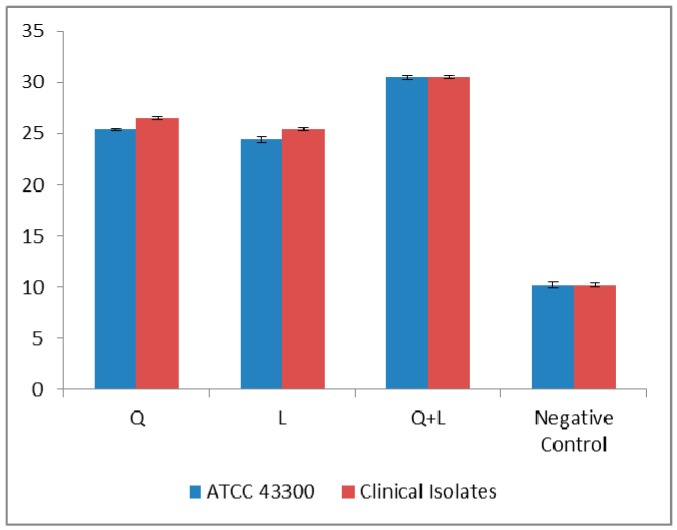
Potassium (K^+^) loss (ppm) caused by Quercetin (Q), luteolin (L) and quercetin + luteolin (Q + L), against MRSA ATCC 43300 strain and MRSA clinical isolates; results are expressed as mean ± standard deviation (*p* < 0.001). Results for Q alone have been previously reported [13].

The damage to the cytoplasm membrane was assessed by potassium leakage for all bacteria (ATCC 43300 strain and clinical isolates) in the case of L, Q [16] and Q + L (*p* < 0.05) (Figure 7).

**Figure 8:**
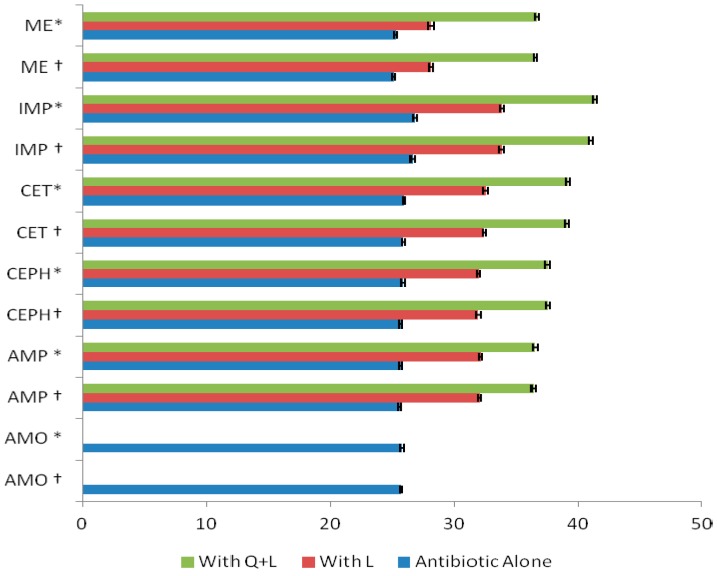
Potassium (K^+^) loss (ppm) caused by luteolin and luteolin + quercetin (L + Q) in combination with antibiotics against * MRSA (methicillin-resistant *Staphylococcus aureus*) clinical isolates (*n* = 100) and † MRSA ATCC 43300strain; results are expressed as mean ± standard deviation (*p* < 0.01 for all combinations apart from AMO).

Cytoplasmic membrane damage was observed for nearly all the antibiotics in combination with flavonoids (Figure 8). In our previous report [16], K^+^ loss in case of AMO was 25.7 ppm for ATCC 43300 strain, while for the clinical isolates the average K^+^ loss was 25.79 ± 0.16 ppm. The highest leakage (26.6 ppm) of potassium was observed for IMP against ATCC 43300, while for clinical isolates it was 26.79 ± 0.14. The K^+^ leakage was higher in the case of Q for ATCC 43300 strain (28.4 ppm) and clinical isolates (28.49 ± 0.14 ppm) compared to L, for which the K^+^ leakage for ATCC 43300 and clinical isolates were 25.4 and 25.49 ± 0.15 ppm, respectively. However, when Q and L were combined, the damage became more pronounced with a leakage of 30.5 and 30.53 ± 0.16 ppm against ATCC 43300 and clinical isolates, respectively, which were higher than the potassium losses caused by Q and L individually. Therefore, antibiotics + flavonoids combination caused a significant potassium loss compared to flavonoids and antibiotics when used alone (*p* < 0.01).

Antimicrobial compounds whose mechanism of action involves microbial cell wall and membrane damage, e.g., phenolic compounds, penicillin, etc., represent a major group. As a consequence, intracellular constituents like proteins and inorganic ions leak from the cells, thus causing irreparable cell damage [22]. Flavonoids have been shown to inflict such damage, such as galangin, which causes significant potassium loss compared to the control and alters cytoplasmic membrane permeability [23]. Similar results were obtained with leucasin, another flavonoid, on *S. aureus* [24].

On the basis of these reports, cell wall damage by flavonoids, antibiotics, and flavonoids + antibiotics was evaluated using the potassium loss assay through flame photometry. To the best of our knowledge, no study has been carried out to evaluate L, L + Q, and their combined effects with conventional antibiotics on cell membrane damage. Our findings suggest that L, L + Q, and their combinations with antibiotics induce cytoplasmic membrane damage, as indicated by the loss of intracellular K^+^, which ultimately compromises the integrity and viability of bacterial cells. As previously mentioned, the antibiotics used in the present study were cell wall inhibitors with a known mechanism of action. The release of K^+^ provides strong evidence that these flavonoids, antibiotics, and their combinations are responsible for damage to the bacterial cell wall, thus explaining their antibacterial activity against MRSA.

In the case of the *mecA* gene sequence comparison in treated and untreated MRSA, after alignment, no mutation was observed. As sequencing of the *mecA* gene in MRSA did not reveal any polymorphism/mutation in the coding region of the gene, there could be a possibility of mutations in the regulatory regions of *mecA*, which results in gene down regulation or non-expression. The mechanism of action of flavonoids, in combination with antibiotics, in the cell wall synthesis inhibition may be due to their effects on different targets.

The size of the *mecA* gene reported was in accordance with the study where a 533-bp *mecA* DNA fragment was amplified in 100% MRSA clinical isolates. This study was carried out to detect the presence of *mecA* in 273 *S. aureus* clinical isolates by PCR [25]. Similarly, in another study, the amplified *mecA* gene product was also 533 bp, used for the identification of MRSA from 94 *S. aureus* clinical isolates collected from both humans and animals in Bangladeshi medical hospitals and veterinary clinics. Basically, the *mecA* gene is present in the methicillin-resistant *Staphylococcus aureus*. This gene within MRSA encodes a specific penicillin-binding protein known as PBP-2a, responsible for antibiotic resistance in *S. aureus*. This is a β-lactam resistant PBP that possesses low affinity for binding with β-lactams [26]. Though the significance of PBP 2 is well documented, its role in cell wall biosynthesis and drug resistance has not yet been defined. The survival and development of *S. aureus* are dependent on both PBP-2 and its transpeptidase portion. The penicillin-binding protein in MRSA is PBP 2a, which is a protein encoded by the *mecA* gene. This PBP 2a acts as a transpeptidase, which is essential for the growth of this bacterium [27,28,29].

Sato and colleagues [30] observed the synergism between β-lactam antibiotics (methicillin, oxacillin, cephaperin, panipenem, streptomycin, ofloxacin, levolfloxacin, vencomycin, erythromycin, kanamycin, and fosfomycin) and flavonoids (flavones and its derivatives), resulting in an increase in the susceptibility of these antibiotics to MRSA. These authors detected a significant reduction in the MIC of the antibiotics investigated. The possible mechanism for this improvement of antibiotic activity by flavonoids may be due to the fact that PBP-2a plays a pivotal role in the cell wall biosynthesis in MRSA [31]. Notably, PBP-2a acts as a bridge in the cross linking of *N*-acetylmuramyl-pentapeptide with pentaglycine, which are two important components of the cell wall. As a result of the synergistic effect of flavonoids and antibiotics, the peptidoglycan synthesis is probably inhibited, which, in turn, reduces the concentration of *N*-acetylmuramyl-pentapeptide or pentaglycine in the developing cell wall. Therefore, PBP-2a would be unable to cross-link the peptidoglycans, thus causing the improvement of antibiotic MIC induced by flavonoids against MRSA. From the present study, it seems that flavonoids may affect the integrity of the bacterial cell wall, as indicated by the loss of potassium ions. The resultant overall cell wall biosynthesis inhibition by flavonoids combined with antibiotics could be the main reason for the enhanced activity of antibiotics.

Another possible mechanism for the synergistic or additive relationship of flavonoids and antibiotics can be the suppression of the efflux pump in MRSA. Kosmidis et al. [32] have identified the existence of eight efflux pumps in MRSA, namely *norA*, *norB*, *norC*, *mdeA*, *qacA/B*, *mepA*, *smr*, *sepA*, and *abcA* [33]. It has been demonstrated that the main expressed efflux genes included *norA*, *norB*, *norC*, *mdeA*, and *mepA* [34], while the resistance to β-lactamantibiotics is due to *abcA* efflux pump. Gibbons et al. showed that the *norA* efflux pump is inhibited by epicatechin gallate (ECG) [35].

It has been observed that monomers (+)-catechin, (−)-ECG, and (−)-epigallocatechin (EGC), in combination with oxacillin, intensify the antibiotic activity against MRSA [36]. The combined effect of these flavonoids was found to be higher than their individual effects. An in vivo study in mice revealed that a combination of (+)-catechin with (−)-ECG greatly reduced the load of bacteria in mice. The effect on bacterial efflux pumps was determined by measuring the daunorubicin (a β-lactam) accumulated in MRSA bacteria found in the infected mice. In addition, the mRNA expression analysis showed a down regulation of the efflux pumps in MRSA. To summarize, (+)-catechin and (−)-ECG increased the activity of β-lactam antibiotics (oxacillin and daunorubicin) against MRSA by down regulating the gene expression of efflux pumps and thus increasing the amount of antibiotics in bacterial cells.

## 3. Experimental Section

### 3.1. Materials

The antibiotic discs were from Oxoid (Hampshire, UK) and included amoxicillin (AMO; 25 µg), ampicillin (AMP; 10 µg), ceftriaxone (CET; 30 µg), cefixime (CEF; 5 µg), cephradine (CEPH; 30 µg), erythromycin (ERY; 15 µg), vancomycin (VAN; 30 µg), methicillin (ME; 10 µg), ciprofloxacin (CIP; 5 µg), levolfloxacin (LEV; 5 µg), sulfamethoxazole-trimethoprim (S-T; 25 µg), and imipenem (IMP; 10 µg). The blank discs were purchased from Himedia (Mumbai, India). Flavonoids luteolin (L) and quercetin (Q), were purchased from Sigma-Aldrich (Irvine, UK), as well as the ethanol used to prepare stock solutions of luteolin (15 g/L) and quercetin (10 g/L). Nutrient agar (NA; CM0003B), Muller Hinton agar (MHA; CM0337B), nutrient broth (NB; CM0001B), and Muller Hinton broth (MHB; CM0405B) were from Oxoid, while mannitol salt agar (MSA; LAB007) was from Lab M Limited (Lancashire, UK).

### 3.2. Bacterial Culturing and Antibacterial Assays

Clinical isolates (*n* = 300) were obtained from the clinical microbiology laboratories of three major tertiary care hospitals of Peshawar, KPK, Pakistan, Hayatabad Medical complex, Lady Reading Hospital, and Khyber Teaching Hospital. The reference strain *S. aureus* (ATCC 43300, Rockville, MD, USA) present in PCSIR laboratories was used as the positive control. All the clinical isolates were sub-cultured on sterile MSA plates at the Department of Microbiology of PCSIR laboratories, Peshawar, Pakistan. Following incubation at 37 ± 1 °C for 18–20 h, plates showing growth were selected and subjected to routine microbiological tests like gram staining, catalase, and coagulase tests [37]. After basic characterization, isolated *S. aureus* were transferred to NB and incubated overnight at 37 °C. Thereafter, the turbidity of bacterial growth was adjusted to 0.5 McFarland using sterile normal saline (0.9% *w*/*v* NaCl in water solution). The standardized inoculum was used to make bacterial lawns on sterile MHA plates subjected to methicillin discs. After overnight incubation at 37 ± 1 °C, plates lacking zones of inhibition around methicillin discs or having <10 mm diameter zones were considered to be methicillin-resistant *S. aureus* (MRSA).

To determine the response of MRSA clinical isolates and ATCC 43300 against luteolin, luteolin + quercetin, antibiotics, and antibiotic + flavonoid combinations, disc diffusion assay was used [38]. Luteolin (L) was tested alone, in combination with quercetin (L + Q) as well as with selected antibiotics (Table 5) at different concentrations. The inhibitory zones were recorded after overnight incubation at 37 °C and all compounds were considered to be active if the inhibitory zones were >10 mm in diameter.

### 3.3. Minimum Inhibitory Concentration (MIC) Assay

A broth macro-dilution method was used for MIC determination of L, L + Q, antibiotics, and antibiotics + flavonoids, at different concentrations (Table 6). Antibiotics showing increased activity in combination with flavonoids included amoxicillin, ampicillin, ceftriaxone, cephradine, imipenem, and methicillin.

An incremental increase approach was adopted to determine the exact MICs of L, L + Q alone, and in combination with antibiotics. In this approach, 20 µg increments were added to the corresponding lower concentrations. Similarly, the MICs of antibiotics alone and in combination with flavonoids against MRSA were also determined to confirm the synergistic or additive effects.

The bacterial cultures were prepared as reported previously and compounds were transferred into sterile tubes. The test tubes containing inoculums and compounds were incubated overnight at 37 °C. The MIC of the compounds was indicated by the absence of any visible growth in test tubes after overnight incubation.

### 3.4. Fractional Inhibitory Concentration (FIC) and FIC Index

To determine the synergistic, antagonistic, or additive effects of flavonoids and antibiotics, FICs were determined using previously described formulas [39]:

FIC of antibiotic (FIC_antibiotic_) = MIC of antibiotic in combination/MIC of antibiotic alone;

FIC of flavonoid (FIC_flavonoid_) = MIC of flavonoid in combination/MIC of flavonoid alone;

FIC Index (FICI) = FIC_antibiotic_ + FIC_flavonoid_.

In case of a combination of two or more flavonoids, FIC were determined using the formula [34]:

FIC = (A/Ma) + (B/Mb)

where A and B are the MICs of compounds in combination, while Ma and Mb were individual MICs of test compounds. The effect of drugs in combination were considered to be synergistic (≤0.5), additive (>0.5 to 1), indifferent (>1 to <2), or antagonistic (≥2) based on previously established criteria [40].

### 3.5. Detection of Cytoplasmic Membrane Damage

The cytoplasmic membrane damage caused by flavonoids and their combinations with antibiotics was determined using a flame photometer (PFP7, Jenway, Gothenberg, Sweden) at a wavelength of 766.480 nm. Instrument calibration and test procedure were in accordance with previously reported method [16].

### 3.6. Modified Ames’s Test

#### 3.6.1. Treatment of Bacterial Cultures with Flavonoids, Antibiotics, and Flavonoids + Antibiotics

MRSA (ATCC 43300) was inoculated aseptically in a nutrient broth and incubated overnight at 37 °C. Thereafter, flavonoids, antibiotics, and their combinations were mixed with 1 mL of overnight grown culture at concentrations lower than their MICs and incubated at 37 °C for 30 min [41]. Genomic DNA from treated cultures was extracted using a bacterial DNA extraction kit (Vivantis Technologies Sdn, Selangor, Malaysia) as per the manufacturer’s instructions. Then, the *mecA* gene was amplified using the extracted DNA as a template for PCR. The following oligonucleotides were used as primers for amplification: Forward Primer (5’AAAATCGATGGTAAAGGTTGGC); Reverse Primer (5’AGTTCTGCAGTACCGGATTTGC). Amplicons from MRSA ATCC 43300 strain treated with flavonoids, flavonoids + antibiotics, and untreated were sequenced using an ABI 3300 (Thermo Fisher Scientific, Waltham, MA, USA) automated sequencer. A consensus sequence of 532 base pairs (*mecA* gene) was obtained by aligning sequences obtained in FASTA, on the basis of forward and reverse primers. The *mecA* gene sequences obtained from untreated cultures were aligned with those from the treated ones, using BioEdit v.7.2.5 (Ibis Biosciences, an Abbott company, Carlsbad, CA, USA).

#### 3.6.2. Statistical Analysis

Results of experiments are expressed as mean ± standard deviation (SD). The data were analyzed by Student’s *t*-test using Statistical Package for Social Sciences (SPSS) version 19, IBM Inc. (Armonk, New York, NY, USA); *p* < 0.05 was considered statistically significant.

## 4. Conclusions

Luteolin exhibited activity against both MRSA ATCC 43300 strain and clinical isolates, and its antibacterial activity was further enhanced when it was used in combination with quercetin. Furthermore, it was observed that luteolin, quercetin, and antibiotics have complemented each another against MRSA. In particular, FICI results indicated additive and synergistic relationships between these flavonoids and some antibiotics, especially the cell wall biosynthesis inhibitors. Potassium loss analysis suggested damage to the cytoplasmic membrane and cell wall. Since no functional variation was detected in the coding sequence of the *mecA* gene, a variation in the regulatory sequence of this gene can be hypothesized, with resultant down regulation or non-expression. The susceptibility of the MRSA bacteria might be due to changes in some other gene sequences putatively involved in the mechanism of multi-drug resistance.

## Figures and Tables

**Table 1:** MIC of luteolin by serial half-dilution and incremental increase method against methicillin-resistant *Staphylococcus aureus* (MRSA) strains.

Method	MRSA ATCC 43300 Strain MIC (µg/mL)	MRSA Clinical Isolates MIC (µg/mL)
Serial half dilution	500	500 (*n* = 39)
		1000 (*n* = 61)
Incremental increase approach	500	516.83 ± 15.67 ^a^
		MIC range: 500–540

^a^ Mean ± standard deviation.

**Table 2:** MICs of luteolin + quercetin (L + Q) by serial half-dilution method and incremental increase approach against methicillin-resistant *Staphylococcus aureus* (MRSA strains).

Method	MRSA ATCC 43300 Strain MIC (µg/mL)	MRSA Clinical Isolates MIC (µg/mL)
Serial half dilution	400 + 200	400 + 200 (*n* = 42)
		800 + 400 (*n* = 58)
Incremental increase approach	380 + 200	(394.06 ± 13.75 ^a^) + (215.04 ± 12.72)
		MIC range: L = 380–420 Q = 200–240

^a^ Mean ± standard deviation.

**Table 3:** Exact MICs by incremental approach of quercetin + luteolin (Q + L) in combination with antibiotics against methicillin-resistant *Staphylococcus aureus* (MRSA) strains.

Antibiotics	Q + L with Antibiotic MIC (µg/mL)
AMO (amoxicillin) ^†^	200 + 380
AMO *	214.06 ± 13.72 ^a^ + 394.06 ± 13.75 ***
MIC * RANGE	Q = 200–240
	L = 380–420
AMP (ampicillin) ^†^	120 + 280
AMP *	134.06 ± 13.85 + 294.06 ± 13.75 ***
MIC * RANGE	Q = 120–160
	L = 280–320
CEPH (cephradine) ^†^	100 + 260
CEPH *	116.06 ± 15.80 + 278.06 ± 15.61 ***
MIC^*^ RANGE	Q = 100–140
	L = 260–300
CET(ceftriaxone) ^†^	80 + 240
CET *	94.06 ± 15.43 + 254.06 ± 14.21 ***
MIC * RANGE	Q = 80−120
	L = 240−300
IMP (imipenem) ^†^	80 + 260
IMP *	97.40 ± 14.49 + 274.06 ± 14.02 ***
MIC * RANGE	Q = 80−120
	L = 260−300
ME(methicillin) ^†^	120 + 300
ME *	134.12 ± 14.95 + 314.06 ± 14.21 ***
MIC * RANGE	Q = 120−160
	L = 300−340

^†^ *S. aureus* ATCC 43300 strain; * Clinical isolates (*n* = 100); ^a^ Mean ± standard deviation; *** *p* < 0.001 compared to flavonoids alone.

**Table 4:** Fractional inhibitory concentration indices (FICI) of flavonoids quercetin (Q), luteolin (L), and antibiotics against MRSA strains.

Flavonoids + Antibiotics	FICI		
	MRSA ATCC 43300	MRSA Clinical Isolates (*n* = 100)	Inference
L + AMP (ampicillin)	0.9	1	Additive
L + CEPH (cephradine)	0.8	1	Additive
L + CET (ceftriaxone)	0.85	1	Additive
L + IMP (imipenem)	0.82	0.9	Additive
L + ME (methicillin)	0.9	1	Additive
Q + L + AMP	0.65	0.68	Additive
Q + L + CEPH	0.60	0.65	Additive
Q + L + CET	0.45	0.50	Synergistic
Q + L + IMP	0.45	0.49	Synergistic
Q + L + ME	0.65	0.69	Additive

**Table 5:** Flavonoid concentrations used in antibiotic sensitivity assays.

Flavonoids	Concentrations Used (µg)
Quercetin	100, 200, 300
Luteolin	100, 200, 300, 400, 500

**Table 6:** Concentrations of flavonoids and their combinations for MIC (minimum inhibitory concentration) assays.

Flavonoids	Concentration Ranges Used for MIC Assays (µg/mL)			
	Broth Half Dilution Assay		Incremental Assay (Exact MIC) ^†^	
	Maximum	Minimum	Maximum	Minimum
Luteolin	1000	125	600	460
Quercetin + Luteolin	400 + 800	100 + 200	320 + 500	180 + 360

^†^ To determine the exact MIC of compounds, an incremental approach was adopted with 20 µg decrease in each dilution.
